# Assessment of Physicochemical Properties and Quality of the Breads Made from Organically Grown Wheat and Legumes

**DOI:** 10.3390/foods13081244

**Published:** 2024-04-18

**Authors:** Grażyna Cacak-Pietrzak, Katarzyna Sujka, Jerzy Księżak, Jolanta Bojarszczuk, Małgorzata Ziarno, Marcin Studnicki, Anna Krajewska, Dariusz Dziki

**Affiliations:** 1 Department of Food Technology and Assessment, Institute of Food Sciences, Warsaw University of Life Sciences, 159C Nowoursynowska Street, 02-776 Warsaw, Poland; grazyna_cacak_pietrzak@sggw.edu.pl (G.C.-P.); katarzyna_sujka@sggw.edu.pl (K.S.); malgorzata_ziarno@sggw.edu.pl (M.Z.); 2 Department of Forage Crop Production, Institute of Soil Sciences and Plant Cultivation—State Research Institute, 8 Czartoryskich Street, 24-100 Pulawy, Poland; jksiezak@iung.pulawy.pl (J.K.); jbojarszczuk@iung.pulawy.pl (J.B.); 3 Department of Biometry, Institute of Agricuture, Warsaw University of Life Sciences, 159C Nowoursynowska Street, 02-776 Warsaw, Poland; marcin_studnicki@sggw.edu.pl; 4 Department of Thermal Technology and Food Process Engineering, University of Life Sciences in Lublin, 31 Głęboka Street, 20-612 Lublin, Poland; anna.krajewska@up.lublin.pl

**Keywords:** legume seeds, chickpeas, field bean, lentil, pea, antioxidant activity, color, texture, quality, sensory analysis

## Abstract

This study aimed to explore the feasibility of substituting wheat flour with varying levels (10%, 15%, 20%, and 25%) of flour derived from field bean, chickpea, lentil, and pea seeds. The investigation focused on assessing the physical properties of wheat dough and the physicochemical characteristics of bread samples. The addition of legume seed flours significantly influenced the dough’s development time, particularly with chickpea flour causing a notable increase in this parameter. While dough stability was generally shorter for mixtures containing wheat flour and legume seed flour, chickpea flour was an exception, significantly prolonging dough stability time. Furthermore, the inclusion of legume flours resulted in increased protein, ash, fiber, fat, and phenolic contents in the enriched bread, while the carbohydrate content decreased. Additionally, the crumb exhibited increased redness and yellowness and decreased lightness due to the enrichment of the bread. Notably, the antioxidant activity of bread containing legume flour also increased, with the most significant increase observed when pea flour was utilized. Conversely, negative effects on bread volume, crumb density, and texture parameters were noted with the incorporation of legume additives. Taking into consideration the results of both physicochemical analyses and sensory evaluation, it is recommended that the incorporation of the specified legume flours should not exceed 15% in relation to the quantity of wheat flour used.

## 1. Introduction

The seeds of leguminous plants constitute a significant component of the human diet in many regions of the world. They have been cultivated since prehistoric times, serving as a traditional source of protein for both humans and animals [1,2]. Regions with the highest consumption of leguminous seeds are Asia and Africa [3]. In recent years, there has been an increase in knowledge regarding the positive impact of leguminous plants on the development of sustainable agriculture and the enhancement of global food security [4,5]. Leguminous crop cultivation is environmentally friendly, primarily due to nitrogen fixation in the soil and low carbon footprint. Leguminous plants exhibit minimal requirements for fertilizers and water, resulting in low production costs [6]. They do not necessitate extensive irrigation and can be cultivated in regions with low precipitation, which is particularly relevant amidst current climate change trends and the increasingly prevalent issue of inadequate rainfall in many parts of the world [7].

Despite the proven benefits associated with the consumption of leguminous seeds, their intake in developed countries remains relatively low. The main reasons identified include divergent dietary habits, low sensory acceptance, and a lack of alternative processed products made from leguminous seeds [5,8]. Due to the amino acid composition of leguminous seed proteins, combining them with cereal ingredients is highly advantageous nutritionally, aiming to enhance the biological value of consumed proteins. In India, many dishes are prepared using both grains and leguminous seeds, such as dhal with rice or chapatti [7]. Furthermore, positive effects have been demonstrated regarding the combination of grains and leguminous seeds on gut microbiota and the associated benefits for colon health and counteracting systemic inflammatory conditions [9].

One method to increase the consumption of leguminous seeds is their incorporation into the formulation of high-consumption products such as bread [10]. Leguminous seeds contain 50% to even 200% more protein than grains [11], which is particularly significant for consumers experiencing protein deficiencies in their diets. Another common practice is the utilization of protein concentrates derived from leguminous seeds. The formulation of many analog products of animal-based foods is based on leguminous plant proteins. To attain desired properties, isolated protein fractions undergo microbiological fermentation or enzymatic hydrolysis [12,13,14]. 

In particular, lactic fermentation is one of the oldest biotechnological processes utilized in food production, including cereal products. Sour dough, made from a blend of rye or wheat flour combined with water, is obtained during spontaneous fermentation or through the use of selected strains of lactic acid bacteria [15]. Positive effects of lactic fermentation have been observed, including improvements in the bioavailability of mineral components, increased solubility of fiber and antioxidant capacity, enhanced protein digestibility, a reduction in the glycemic index, and increased contents of phenolics [16,17]. Additionally, the beneficial impact of lactic fermentation on reducing the content of antinutritional factors in products derived from leguminous seeds has been demonstrated [18,19].

The aim of the study was to assess the suitability of flour derived from selected leguminous plants, such as *Cicer arietinum* (chickpea), *Lens culinaris* (lentil), *Pisum sativum* L. (pea), and *Vicia faba* L. (field bean), as a component used in creating wheat sourdough bread. The flour from these seeds was used due to their mentioned beneficial agricultural and nutritional characteristics.

## 2. Materials and Methods

### 2.1. Materials

Bread dough was prepared using organic basic raw materials (organic wheat flour with protein content 11.12% DM (dry mass), ash content 0.54% DM, wet gluten content 24.6%, and falling number 298 s). Yeast and salt were also used [10]. Moreover, organic flour from chickpeas (CPs), field beans (FBs), lentils (Ls), and peas (Ps) were also utilized. Legume seeds were sourced from the Osiny Experimental Station, affiliated with the Institute of Soil Science and Plant Cultivation—State Research Institute in Pulawy (Poland). The *Lactiplantibacillus plantarum* and *Levilactobacillus brevis* cultures used originated from the Pure Culture Collection of the Department of Milk Technology at the Warsaw University of Life Sciences (WULS) in Warsaw (Poland). After purification, legume seeds were ground into particles below 1.0 mm [10]. Chemical reagents utilized in the research were of analytical-grade purity and included, sodium salicylate, gallic acid, methanol, DPPH, and ABTS. Reagents of analytical grade purity were procured from Sigma-Aldrich (Poznań, Poland).

### 2.2. Baking Parameters of Raw Materials

In the analysis of wheat flour, the falling number, gluten yield, and quality were assessed [20]. Additionally, the rheological characteristics of wheat flour using a Farinograph were evaluated. The methods employed for these tests were previously described in the study by Cacak-Pietrzak et al. [10].

### 2.3. Bread Preparation

The dough was made by employing a two-stage process, beginning with a 200% hydration sourdough initiated with *Lactiplantibacillus plantarum* and *Levilactobacillus brevis* bacteria cultures. The sourdough fermentation was carried out over a 7-day period at 25 °C to develop specific characteristics. The control sample consisted of wheat flour (700 g), sourdough (constituting 10% relative to the flour mass), 21 g of fresh compressed yeast (making up 3% relative to the flour mass), and 10.5 g of salt (accounting for 1.5% relative to the flour mass). In the experimental variations, wheat flour was systematically replaced with flour derived from leguminous seeds, with replacement levels set at 10%, 15%, 20%, and 25%. A detailed description of the bread-making process has been provided by Cacak-Pietrzak et al. [10].

### 2.4. Analysis of the Basic Chemical Composition

The moisture content, total ash content, fiber content, total protein content, fat content, and carbohydrate content were determined. The fundamental chemical compositions of both the raw materials and the bread were analyzed using AACC methods [20]. Detailed information about the methods employed was provided by Cacak-Pietrzak et al. [10].

### 2.5. Analysis of Bread Physical Parameters

After 24 h from baking, the breads were weighed; the bread yield and total baking loss were calculated; and then the loaf volume of bread, crumb density, and texture parameters were determined [21].

### 2.6. Analysis of Raw Materials and Bread Color

The color parameters of samples (raw materials and bread) were determined using the reflection method in the CIE-Lab* system with the CR-200 colorimeter (Konica Minolta, Osaka, Japan) and the absolute color difference (ΔE*) was calculated [22].

### 2.7. Analysis of Polyphenol Content and Antioxidant Capacity

The total polyphenol content and the ability to quench DPPH free radicals and ABTS•+ cation radicals were determined in raw materials as well as in bread.

#### 2.7.1. Preparation of Extracts

Methanolic extracts of raw materials and bread were prepared according to the method outlined by Krajewska et al. [23].

#### 2.7.2. Total Polyphenol Content (TPC)

TPC was determined using the Folin–Ciocalteau spectrophotometric method [24] with modification presented by Krajewska et al. [23] and expressed in mg of GAE (gallic acid equivalents) per g of dry mass (DM).

#### 2.7.3. DPPH and ABTS•+ Scavenging Activity

The assessment of the capacity to neutralize DPPH free radicals was conducted using a spectrophotometric method outlined by Brand-Williams et al. [25]. The capacity to quench ABTS•+ cation radicals was evaluated using a spectrophotometric method as detailed by Re et al. [26]. The ability to quench radicals was expressed as EC_50_ [23]. A stronger antioxidant activity is indicated by a lower EC50 value because it signifies that a smaller amount of the antioxidant is required to achieve the intended outcome [10].

Total polyphenols and antioxidant activity were assessed using the spectrophotometer, as referenced in Krajewska et al. [23].

### 2.8. Sensory Analysis of Bread

The sensory evaluation of bread (9-point hedonic test) was conducted 24 h after baking following the methodology outlined by Garcia-Gómez et al. [27]. The assessment panel consisted of 56 members (employees and students of the Warsaw University of Life Sciences—WULS) aged between 21 and 60 years.

### 2.9. Statistical Analysis of Results

The statistical analysis of the results involved a minimum of three repetitions for all measurements. R software version 4.3.0 [28] was employed for the analysis. Analysis of variance (ANOVA) served as a post hoc test, utilizing Tukey’s multiple comparison procedure. Identical lettering was used to indicate no significant differences at a significance level of α = 0.05.

Moreover, to explore the relationship between the characteristics of the investigated bread and the type and level of added flour from legume seeds, cluster analysis and Principal Component Analysis (PCA) were conducted. These additional analyses aimed to provide a comprehensive understanding of the variables and their interrelationships in the study.

## 3. Results

### 3.1. Water Absorption and Dough Characteristic

The water absorption (WA) of wheat flour was 54.2%. The values of this parameter for wheat flour blends with flour from legume seeds ranged from 53.1% (CP10) to 56.2% (L25) (Table 1). Substituting wheat flour with CP flour up to 20% and with FB flour up to 25% had no significant influence on the WA of the blends. However, substituting wheat flour with L and P flours caused an increase in WA, but this increase was statistically significant only in the case of the L25 sample. The development time of the control dough (CD) was 1.9 min. The development times of dough from blends of wheat flour with legume seed flours ranged from 5.8 min (FB25, P20) to 10.9 min (CP15) and were notably longer compared to those of the t control sample. The stability time of the control dough (CD) was 9.0 min. Substituting wheat flour with CP flour, regardless of the level of addition, significantly prolonged the dough stability time (14.7–18.8 min). Interestingly, in the case of adding flours from other legume seeds, the dough stability times were shorter, and, except for the P10 sample, these changes were statistically significant. The softening of CD was 36 FU. Substituting wheat flour with legume seed flours generally resulted in a significant decrease in dough softening, except for the addition of FB flour at levels of 20% and 25%, where dough softening significantly increased compared to the CD.

### 3.2. Basic Properties of Bread

The bake loss of the control wheat bread (C) registered at 11.5% (as indicated in Table 2). In bread formulations where a portion of the wheat flour was substituted with legume seed flour, bake loss varied from 11.0% (CP20, CP25, and L15) to 12.7% (CP10), exhibiting irregular fluctuations in this parameter. The yield of the control bread amounted to 139.2%, whereas bread incorporating legume seed flour ranged from 135.9% (CP10) to 140.3% (L25). A statistically notable enhancement in bread yield was observed following the inclusion of CP and L at the 25% level, accompanied by a decrease after incorporating CP at the 10% level; otherwise, bread yield remained akin to the control sample. The volume of (C) measured 368 cm^3^ per 100 g, with a crumb density of 0.25 g cm^−3^. The addition of legume seed flour precipitated a substantial decrease in bread volume, resulting in an elevation in crumb density. These parameters exhibited a linear correlation with the escalating degree of substitution. The most significant reduction in volume (320–247 cm^3^ per 100 g) and elevation in crumb density (0.32–0.44 g cm^−3^) were noted upon substituting wheat flour with lentil flour.

### 3.3. Crumb Texture

The crumb hardness of the control sample (C) was measured at 8.16 N (Table 3). Substituting wheat flour with legume flour significantly influenced the increase in crumb hardness of bread in a statistically significant manner. As the level of fortification increased, the values of this parameter exhibited a linear increase. The most significant increase in hardness (12.68–20.40 N) compared to the control sample occurred when substituting wheat flour with lentil flour. The elasticity, springiness, and cohesiveness of the crumb of the control bread were 0.23, 0.88, and 0.66, respectively. The addition of legume flour resulted in a consistent decrease in the values of certain parameters, following a linear trend. However, it is noted that these changes did not always reach statistical significance.

### 3.4. Color Coordinates

The lightness (L*) of the control crumb was 71.22 (Table 4). Substituting wheat flour with flour from legume seeds significantly affected the decrease in crumb lightness. As the proportion of legume flour incorporated escalated, there was a consistent linear decline observed in the values of this parameter. The most substantial changes in the lightness (60.49–52.90) sample occurred when lentil flour was added into the bread recipe. The color parameters of redness and yellowness of the control bread were 0.12 and 13.64, respectively. Substituting wheat flour with flour from legume seeds significantly influenced the increase in the values of these color parameters of bread crumb. Bread enriched with lentil flour exhibited a particularly high intensity of redness (R*) (1.26–2.46), while bread with chickpea flour addition showed a high intensity of yellowness (16.87–22.65). The absolute color difference (ΔE) between the control bread and the legume-flour-enriched bread ranged from 3.0 to 18.4. This indicates that adding flour from all the legume seeds used in the experiment, even in quantities as low as 10%, had a significant impact on the color of the bread crumb, and the changes in terms of darkening were noticeable even to an inexperienced observer.

### 3.5. Basic Chemical Composition

The control bread contained 84.54% DM carbohydrates, 11.39% DM protein, 0.86% DM total ash, 1.91% DM dietary fiber, and 1.39% DM fat. Replacing wheat flour with legume seed flour led to a statistically notable reduction in carbohydrate content and a rise in protein, fiber, and total ash content in the bread, as indicated in Table 5. Except for the samples with lentil flour addition, there was also an increase in the fat content in the bread. Changes in the nutrient content in bread enriched with flour from legume seeds were attributed to differences in the chemical composition of wheat flour and individual flours from legume seeds. Flours from legume seeds were notably characterized by high protein content (22.18–36.32% DM), 2–3 times higher than wheat flour (11.12% DM). They also contained significantly more ash than wheat flour (respectively, 2.86–4.08% DM and 0.54% DM) and dietary fiber (respectively, 5.40–7.77 and 1.85% DM). The fat content in wheat flour was 0.85% DM, whereas in flours from legume seeds it ranged widely from 0.39% DM (lentil) to 4.88% DM (chickpea). The chemical composition of bread changed linearly with increasing levels of the addition of flour from legume seeds.

### 3.6. Phenolics Content and Antioxidant Activity

Among the raw materials used in the study, wheat flour exhibited the lowest content of phenolic compounds (0.45 mg GAE g DM^−1^), while the highest level of polyphenols was observed in pea flour (2.36 mg GAE g DM^−1^) (Table 6). These results were well correlated with antioxidant activity. Both the highest activity against DPPH and ABTS radicals (the lowest EC_50_ values and, consequently, the strongest antioxidant activity) were observed for pea flour, while the lowest antioxidant activity was found for wheat flour. The varying content of phenolics in the legume flours resulted in significant differences in their content in bread. The highest amount of phenolic compounds (0.77–1.37 mg GAE g DM^−1^), along with the highest antioxidant activity against both DPPH and ABTS, was detected in bread with the addition of pea flour. Conversely, wheat bread enriched with chickpea flour exhibited significantly lower phenolic content compared to other loaves where part of the wheat flour was replaced with pea, field bean, and lentil flour. This directly translated into the antioxidant activity of the bread. Both for ABTS and DPPH antioxidant activity, the lowest EC_50_ values (lowest antioxidant activity) were observed for bread enriched with chickpea flour, and the highest values of this index were found for products where part of the wheat flour was replaced with pea flour.

### 3.7. Sensory Attributes of Bread Samples

**Figure 1:**
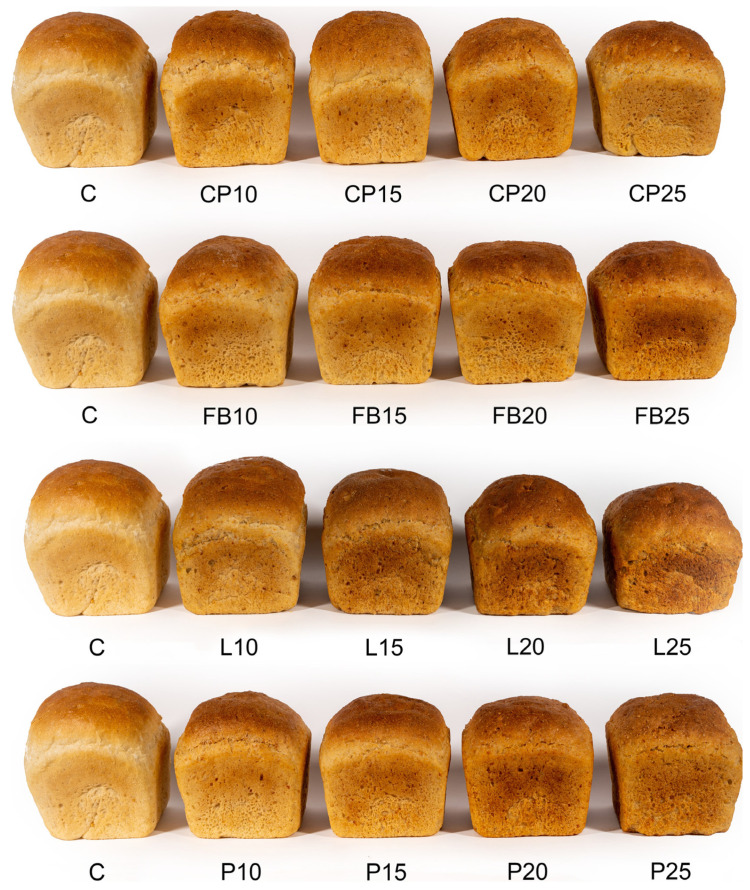
Appearance of bread loaves. C—control bread, CP10–CP25—breads containing, respectively, 10, 15, 20, and 25% chickpea flour, FB10–FB25—breads containing, respectively, 10, 15, 20, and 25% field bean flour; L10–L25—breads containing, respectively, 10, 15, 20, and 25% lentil flour; P10–P25—breads containing, respectively, 10, 15, 20, and 25% pea flour.

When evaluating the sensory attributes of the bread, panelists considered factors such as its shape, degree of expansion, and the visual characteristics of the crust. The control sample received the highest scores (8.5 points) for these attributes, indicating the most pronounced loaf expansion (refer to Table 7 and Figure 1). Comparable scores were awarded to sample L10. Generally, the replacement of wheat flour with flour derived from legume seeds caused a significant reduction in loaf expansion, particularly noticeable at higher levels of this additive. However, the panelists had no major reservations regarding the loaf shape, which was appropriate for the form in which dough portions were baked, and the visual characteristics of the crust. Therefore, scores for the appearance of the loaf exceeded five points on a nine-point hedonic scale, indicating acceptance above the established threshold of consumer acceptability. High scores were also awarded for the taste and aroma of the bread. The control bread obtained the highest scores in these regards, garnering 8.5 and 8.7 points, respectively, being the most delicately flavored, slightly acidic, and exceptionally aromatic. As the proportion of flour from legume seeds increased, scores for these attributes gradually decreased. At a 25% inclusion level of chickpea, pea, and lentil flour, as well as 20% and 25% inclusion levels of pea flour, scores for aroma and taste fell below the threshold of consumer acceptability.

**Figure 2:**
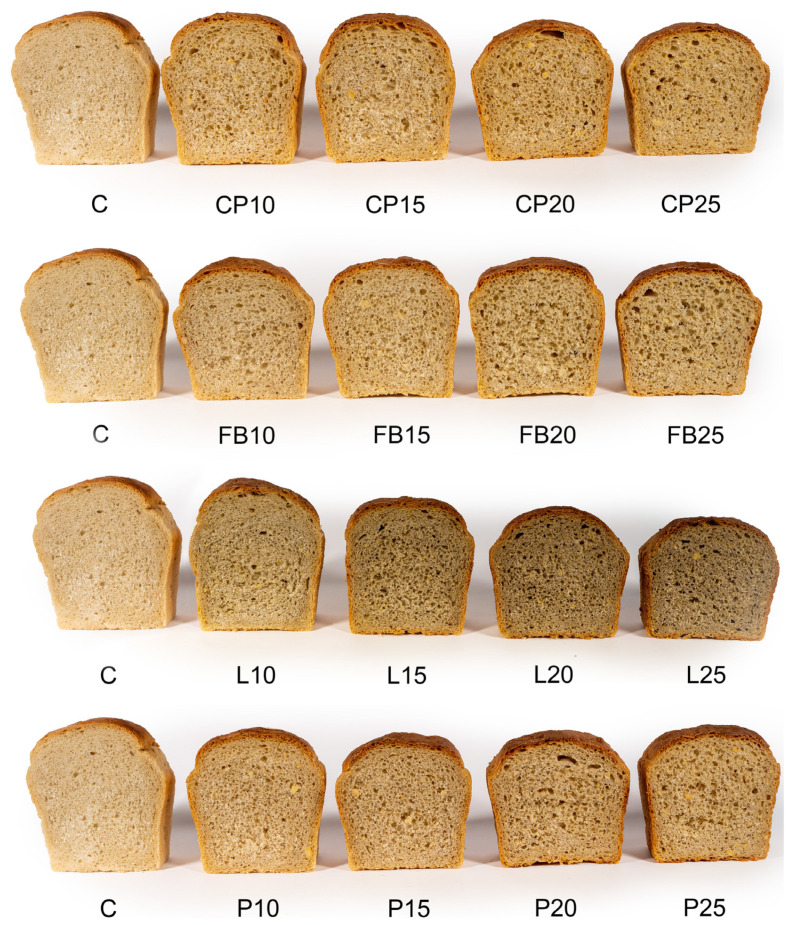
Bread crumb appearance. C—control bread, CP10–CP25—breads containing, respectively, 10, 15, 20, and 25% chickpea flour, FB10–FB25—breads containing, respectively, 10, 15, 20, and 25% field bean flour; L10–L25—breads containing, respectively, 10, 15, 20, and 25% lentil flour; P10–P25—breads containing, respectively, 10, 15, 20, and 25% pea flour.

When assessing the texture, the control bread and sample L10 received the highest scores from the panelists, garnering 8.7 and 8.3 points, respectively (Table 7). These samples exhibited a fine-pored and highly homogeneous crumb structure, as illustrated in Figure 2. As the quantities of legume flour added increased, the texture of crumb became denser, displaying larger irregular pores on the cross-section (Figure 2). In alignment with taste and aroma evaluations, bread incorporating 25% chickpea, field bean, and lentil flour, as well as 20% pea flour, fell below the threshold of consumer acceptability.

Regarding color, the control bread received the highest score of 8.1 points, closely followed by samples CP10, FB10, and P10, which scored between 8.0 and 8.1 points. These samples featured a light brown crust color and beige crumb color. The introduction of legume flour led to a darkening of both crust and crumb, particularly noticeable in the case of bread with lentil flour. The darkened color resulted in bread with 25% chickpea, field bean, and lentil flour, as well as 20% pea flour, being rated below the threshold of consumer acceptability.

Considering the overall sensory scores provided by the panelists, it was deduced that the maximum inclusion of chickpea, field bean, and lentil flour should not exceed 20%, and pea flour should not exceed 15%.

### 3.8. Cluster Analysis (CA) and Principal Component Analysis (PCA)

**Figure 3:**
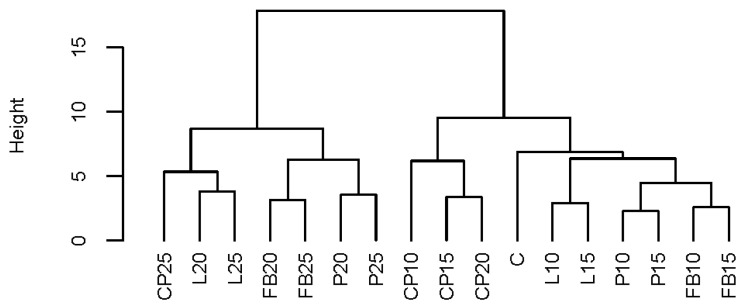
The results of cluster analysis. C—control bread, CP10–CP25—breads containing, respectively, 10, 15, 20, and 25% chickpea flour, FB10–FB25—breads containing, respectively, 10, 15, 20, and 25% field bean flour; L10–L25—breads containing, respectively, 10, 15, 20, and 25% lentil flour; P10–P25—breads containing, respectively, 10, 15, 20, and 25% pea flour.

The outcomes of the CA are depicted in Figure 3. This analysis facilitated the identification of two distinct groups within the experimental combinations. The first group exhibited similarity among bread samples with the inclusion of each type of legume flour at levels of 10% and 15%. Conversely, the second group showcased resemblance among bread samples incorporating legume flour at levels of 20% and 25%. Notably, at lower levels of this additive, the bread parameters closely resembled those of traditional wheat bread, as observed in the control sample.

**Figure 4:**
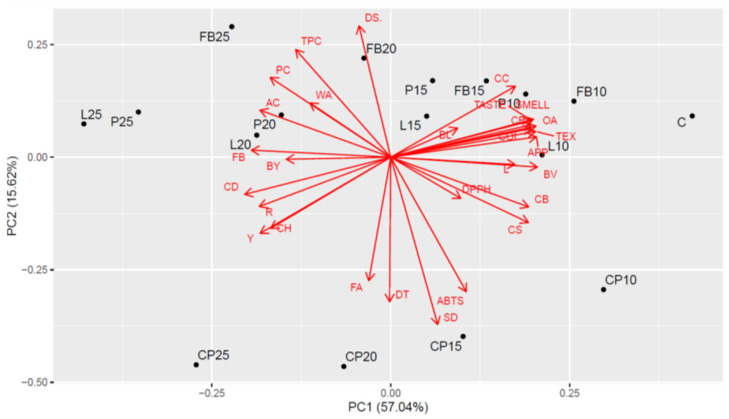
Biplot of Principal Component Analysis (PCA) for all examined characteristics. C—control bread; CP10–CP25—breads containing, respectively, 10, 15, 20, and 25% chickpea flour, FB10–FB25—breads containing, respectively, 10, 15, 20, and 25% field bean flour; L10–L25—breads containing, respectively, 10, 15, 20, and 25% lentil flour; P10–P25—breads containing, respectively, 10, 15, 20, and 25% pea flour; WA—water absorption; DT—development time; SD—stability of dough; DS—degree of softening; BL—baking loss; BY—bread yield; BV—bread volume; CD—crumb density; CH—hardness; CE—elasticity; CS—springiness; CC—cohesiveness; L—lightness; R—redness; Y—yellowness; APP—appearance; TEX—texture; COL—color; OA—overall acceptability; PC—protein; AC—ash; FB—fiber; FA—fat; CB—carbohydrates.

The outcomes of the Principal Component Analysis (PCA) are illustrated in Figure 4. The PCA uncovered robust relationships among the sensory parameters evaluated in the bread samples. Specifically, bread with lower levels of legume flour addition (10% and 15%) and the wheat bread (control sample) exhibited elevated values for these parameters. The contents of PC (Preservation Capacity), AC (Aroma Complexity), TPC (Taste Perceived Complexity), and WA (overall acceptability) were strongly and positively correlated with each other, with peak values observed in combinations featuring the highest inclusion of legume flour at 25%. Notably, high values of the parameters SD (Sensory Desirability), DT (Dough Texture), and ABTS (antioxidant activity) were noted for bread with the addition of chickpea flour (CP) across all of its inclusion levels.

## 4. Discussion

Consumers are increasingly paying attention to the composition of food and seeking products with high nutritional potential and a high content of health-promoting components (antioxidants, vitamins, and minerals). One of the staple foods in the human daily diet is bread. Due to its simple composition and frequency of consumption, bread serves as a good matrix for designing food with health-promoting properties. Studies conducted in many laboratories indicate that valuable ingredients for bread formulation can include, among others, flours from non-cereal grains and pseudocereals [16,29,30], herbs and spices [31,32], bran [33,34,35], as well as flours from legume seeds [8,10,36]. These introduced ingredients influence the physicochemical and sensory properties of the final product, thus it is necessary to individually determine the optimal, consumer-acceptable level of a particular type of additive. They also affect the course of the production process, including the yield and rheological properties of the dough [10,36,37]. In our study, the substitution of wheat flour with flour from four species of legume plants—chickpea, field bean, lentil, and pea—did not significantly affect the water absorption of the blends. Variations in the water absorption of flour blends can stem from differences in the chemical composition of the additives used and the interaction between wheat flour and legume flour. Specifically, the higher fiber content found in legume seed flour compared to wheat flour could potentially increase the water absorption of flour blends. On the other hand, substituting wheat flour in the recipe may lead to a decrease in the water absorption of flour blends due to a reduction in the amount of gluten, which also significantly affects water absorption [10]. The literature data [10,36] indicate that wheat flour blends with legume flour generally exhibit higher water absorption than wheat flour alone, due to the higher content of water-absorbing components such as protein and dietary fiber than in wheat flour. There are also available studies [37] indicating that the addition of green lentil flour to wheat flour reduced its water absorption. In our study, the water absorption of wheat flour blends with legume flour was at a similar level, and a statistically significant increase in water absorption compared to the control sample was observed only in the L25 sample. However, the legume seeds caused significant changes in the rheological properties of the dough. Similar to previous studies [10], regardless of the type and level of the additive used, there was a significant extension of the dough development time, which was probably due to dietary fiber, which hinders the formation of the dough gluten matrix [21]. An extension of the dough development time with the addition of lentil flour was also achieved by Turfani et al. [37]. Depending on the type of additive used, the dough stability time was shortened (FB, L, and P) or extended (CP). Doughs with CP flour also stood out for their low level of softening, 5–6 times lower compared to wheat dough. The usual duration of dough stability, as assessed via the farinograph when using wheat flour, typically falls between 6 to 10 min. However, this timeframe may fluctuate based on various factors including the flour’s quality, protein concentration, and other variables associated with its specific composition, extraction rate, and the baking procedure [38]. The increased stability of chickpea-enriched dough can be explained by its exceptionally high fat and fiber content, which are over five and three times higher, respectively, than those in wheat flour. A similar trend was found when we previously added yellow lupine flour into wheat dough with 6.42% fat content and 19.31% fiber content [10]. The stability and softening of dough also significantly depend on its fiber content. However, the impact of fiber on dough softening can be complex and may depend on various factors such as the type and quantity of fiber, type of flour, additional ingredients, etc.; as a result, fiber can both increase and decrease dough softening [10,39].

The addition of flour from other legume seeds generally also had a beneficial effect on dough softening, except for samples FB20 and FB25. However, the substitution of wheat flour with flour from legume seeds did not significantly affect the yield of the resulting bread, which can be explained by the small variation in farinographic water absorption of wheat flour and the blends made from it. The bread yield was relatively low, except for the L25 sample, it did not exceed 140%. This was due to the low water absorption of the organic wheat flour used for baking, typical for light wheat flours from organic production [10].

The incorporation of legume flour into the recipe resulted in changes in the physical parameters of the loaves. Regardless of the type and level of addition, a substantial reduction in loaf volume occurred, leading to an increase in bulk density and crumb hardness. Additionally, a decrease in crumb elasticity and springiness was observed. These parameters exhibited a linear trend with increasing levels of legume flour, with the most pronounced differences observed in bread supplemented with lentil flour, characterized by an exceptionally low fat content. The adverse effect of adding legume seed flour on loaf volume was also noted in the studies conducted by Turfani et al. [37], consistent with the decrease in volume and deterioration in porosity and texture parameters of bread crumb observed in our earlier research [10]. This occurrence can be explained by the elevated levels of dietary fiber and non-gluten proteins present in legume seed flours. The presence of these components hinders the formation of the gluten network, contributing to its weakening [21,40,41]. The development of a less robust gluten network results in increased loss of carbon dioxide during fermentation, leading to poorer dough expansion. Consequently, bread with decreased volume is obtained [42,43], as well as adversely affecting bread crumb properties [10]. Importantly, the increased stability of dough with CP did not positively correlate with the volume of the resulting breads, as commonly observed in the case of wheat flour [44]. When various plant additives are incorporated into wheat flour, the stability of the wheat dough can be extended. However, this does not always correspond with an increase in bread volume. Sometimes, despite increased dough stability, the volume of the bread decreases. This could be due to negative consequences related to dough weakening during fermentation. Similar tendencies were observed by other authors when incorporating yellow lupine flour into wheat dough [10].

Substitution of wheat flour with legume seed flour also significantly affected the crumb color of the bread. Compared to the control, a decrease in brightness and an increase in the proportion of red and yellow hues were observed, attributable to the presence of natural pigments, primarily carotenoids, with intensely yellow–orange coloration [45,46]. Similar to previous studies [10], darkening of the crumb color occurred linearly with increasing incorporation of legume seed flours into the bread recipe, becoming noticeable even at the lowest level of addition used in the experiment (10%), discernible even to an inexperienced observer (ΔE values > 2).

The introduction of flour from legume seeds into the recipe positively impacted the nutritional value of bread, reducing carbohydrate content in favor of increased protein, dietary fiber, mineral content, and, with the exception of lentil-added trials, fat. It is noteworthy that, in addition to significantly increasing the overall protein content in bread, the addition of legume seed flours contributed to enhanced protein digestibility. Legume plant proteins mainly consist of albumins and globulins, which have a more favorable amino acid composition than wheat proteins [47,48]. They are good sources of amino acids such as arginine, leucine, aspartic acid, and glutamic acid [1,7], as well as lysine, which is a limiting amino acid in wheat protein [48]. Increasing the dietary fiber and mineral content in bread is also nutritionally advantageous. Compared to wheat bread (C), bread with a 25% addition of legume seed flour contained approximately twice as much dietary fiber and 1.5–1.7 times more minerals. Legume seeds are rich sources of macroelements such as Ca, Mg, K, and Na, and microelements such as Cu, Zn, and Mn [49,50]. The fiber consists mainly of insoluble fractions, which constitute about 75% of the total fiber content [46].

When developing recipes and production technology for new bakery products, it is essential to consider that, in addition to high nutritional value, they must be acceptable to consumers in terms of sensory properties. The consumers’ acceptability of bread depend on the raw materials used and the process parameters [51]. In our study, a two-phase method was used to prepare the bread dough. The first phase involved a previously prepared wheat sourdough inoculated with cultures of *Lactiplantibacillus plantarum* and *Levilactobacillus brevis* bacteria. This method, due to the long sourdough maturation time and associated higher costs, is rarely used for wheat bread production in industrial conditions, despite its ability to produce bread with health-promoting properties. These properties mainly result from the presence of lactic acid produced during fermentation, which regulates the pH of the gastrointestinal tract and contributes to proper digestion and elimination processes [52,53]. Additionally, lactic fermentation reduces the activity of protease inhibitors, increasing protein digestibility. It also eliminates other antinutritional factors, including phytates, which limit the bioavailability of macro- and microelements [49,54]. Moreover, lactic fermentation can be an effective way to reduce the immunoreactivity of the product, facilitated by the higher activity of protein-hydrolyzing enzymes in acidic environments, including gluten proteins [55].

White wheat flour is characterized by a low content of phenolic compounds, which, consequently, results in low antioxidant activity. The utilized legume seed flours exhibited significantly higher levels of phenolic compounds and greater ability to scavenge ABTS and DPPH radicals. This led to an increase in the content of bioactive compounds in bread as well as an increase in antioxidant activity. Among the employed legume seed flours, the best effect in terms of phenolic compound content and increased antioxidant activity was observed when wheat flour was replaced with pea flour. Conversely, chickpea flour had a relatively minor impact on these bread parameters. Other authors also observed a similar effect when grass pea and lupine seed flour were added to wheat bread [10].

Bread made with sourdough is characterized by better taste and aroma as well as longer shelf life than wheat bread produced directly with yeast [52,56]. In our studies, sourdough bread with legume flour achieved high consumer acceptance. Taking into account the overall ratings given by the panelists for the sensory properties of bread, the maximum inclusion levels of chickpea, pea, and lentil flour in the recipe were determined to be 20%, while pea flour was set at 15%. These determined high levels of legume seed flour inclusion ensure the production of loaves with enhanced nutritional value and simultaneously acceptable sensory characteristics.

## 5. Conclusions

Legume seed flour impacted both the physical properties of the wheat dough and the physicochemical attributes of the resulting bread. The introduction of legume seed flours did not notably alter the water absorption of the dough mixtures when compared to the control. However, significant differences were observed in both development time and stability time, particularly when chickpea flour was used. This confirms that legume seed flours may interact with the wheat flour dough in various ways. Additionally, the conducted studies demonstrated the favorable impact of adding legume seed flours on the chemical composition of bread, especially the protein content (field bean), fiber (lentil and pea), mineral content (lentil), and polyphenols (pea and field bean). The protein content increased from the level of 11.39% DM for the control bread to 18.22% DM, 16.56% DM, 16.09% DM, and 14.47% DM for the FB25, P25, L25, and CP25 samples, respectively. Furthermore, the enriched bread showed increased antioxidant activity. This effect was most pronounced when the bread was enriched with pea flour. Negative effects of the additives were observed, particularly in terms of bread volume, crumb density, crumb hardness, and consumer acceptance. Moreover, the inclusion of legume flours caused a decrease in crumb brightness, as well as increased redness and yellowness. Cluster analysis identified two main groups based on the addition of legume flour: loaves with 20% and 25% of legume flour and the remaining bread variants (10% and 15% addition), which were most similar to the control. Additionally, PCA revealed a strong association between the sensory parameters and the tested bread samples. Taking into account the results of the sensory evaluation, the addition of the considered legume flours should not exceed 15% in relation to the wheat flour used.

## Figures and Tables

**Table 1:** Farinograph properties of dough.

SA	WA [%]	DT [min]	SD [min]	DS [FU]
CD	54.2 ± 0.12 ^ab^	1.9 ± 0.05 ^a^	9.0 ± 0.12 ^e^	36 ± 2.05 ^d^
CP10	53.1 ± 0.12 ^a^	10.4 ± 0.21 ^f^	18.8 ± 0.08 ^g^	5 ± 0.82 ^a^
CP15	53.2 ± 0.08 ^a^	10.9 ± 0.29 ^f^	15.1 ± 0.08 ^f^	5 ± 0.00 ^a^
CP20	53.7 ± 0.12 ^ab^	10.4 ± 0.24 ^f^	14.9 ± 0.12 ^f^	6 ± 2.62 ^a^
CP25	54.6 ± 0.19 ^b^	9.2 ± 0.00 ^e^	14.7 ± 0.42 ^f^	5 ± 0.94 ^a^
FB10	53.8 ± 0.12 ^ab^	8.0 ± 0.21 ^d^	7.6 ± 0.33 ^d^	21 ± 0.47 ^bc^
FB15	53.4 ± 0.05 ^a^	5.9 ± 0.12 ^b^	6.9 ± 0.29 ^cd^	27 ± 0.47 ^c^
FB20	53.6 ± 0.05 ^a^	6.1 ± 0.09 ^bc^	4.4 ± 0.12 ^b^	57 ± 2.05 ^e^
FB25	53.7 ± 0.05 ^ab^	5.8 ± 0.26 ^b^	3.0 ± 0.24 ^a^	85 ± 0.47 ^f^
L10	55.0 ± 0.05 ^b^	8.2 ± 0.05 ^d^	8.2 ± 0.00 ^d^	20 ± 2.05 ^b^
L15	55.5 ± 0.05 ^b^	7.2 ± 0.05 ^d^	6.4 ± 0.14 ^cd^	24 ± 1.25 ^c^
L20	55.6 ± 0.17 ^bc^	6.8 ± 0.05 ^c^	6.1 ± 0.12 ^c^	25 ± 2.05 ^c^
L25	56.2 ± 0.08 ^c^	6.0 ± 0.05 ^bc^	5.7 ± 0.25 ^c^	30 ± 2.36 ^d^
P10	55.1 ± 0.00 ^b^	6.5 ± 0.16 ^c^	9.5 ± 0.57 ^e^	20 ± 2.05 ^b^
P15	55.1 ± 0.05 ^b^	6.5 ± 0.00 ^c^	8.2 ± 0.33 ^d^	21 ± 0.82 ^bc^
P20	55.5 ± 0.00 ^b^	5.8 ± 0.54 ^b^	8.1 ± 0.09 ^d^	16 ± 1.25 ^b^
P25	55.7 ± 0.05 ^bc^	6.2 ± 0.00 ^bc^	6.8 ± 0.12 ^cd^	25 ± 0.00 ^c^

SA—sample, WA—water absorption, DT—development time, SD—stability of dough, DS—degree of softening, CD—control dough, CP10–CP25—dough containing, respectively, 10, 15, 20, and 25% chickpea flour, FB10–FB25—dough containing, respectively, 10, 15, 20, and 25% field bean flour; L10–L25—dough containing, respectively, 10, 15, 20, and 25% lentil flour; P10–P25—dough containing, respectively, 10, 15, 20, and 25% pea flour; the values designated by the letters ^a–g^ are significantly different (*p* < 0.05).

**Table 2:** Baking loss, bread yield, and basic properties of bread samples.

SA	BL [%]	BY [%]	BV [cm^3^ 100^−1^ g]	CD [g cm^−3^]
C	11.5 ± 0.05 ^b^	139.2 ± 0.74 ^b^	368 ± 3.74 ^f^	0.25 ± 0.01 ^a^
CP10	12.7 ± 0.09 ^d^	135.9 ± 0.36 ^a^	335 ± 3.09 ^e^	0.32 ± 0.00 ^b^
CP15	11.8 ± 0.12 ^b^	137.7 ± 0.29 ^ab^	321 ± 2.62 ^d^	0.33 ± 0.01 ^c^
CP20	11.0 ± 0.21 ^ab^	139.4 ± 0.37 ^bc^	298 ± 1.70 ^c^	0.38 ± 0.01 ^d^
CP25	11.0 ± 0.29 ^ab^	140.0 ± 0.50 ^c^	280 ± 1.70 ^ab^	0.42 ± 0.01 ^e^
FB10	12.1 ± 0.12 ^c^	137.6 ± 0.25 ^ab^	326 ± 4.64 ^e^	0.32 ± 0.00 ^b^
FB15	11.9 ± 0.22 ^b^	137.9 ± 1.10 ^ab^	316 ± 4.97 ^d^	0.34 ± 0.01 ^c^
FB20	12.1 ± 0.17 ^c^	137.8 ± 1.19 ^ab^	308 ± 2.62 ^cd^	0.35 ± 0.00 ^cd^
FB25	12.0 ± 0.25 ^bc^	137.9 ± 0.64 ^ab^	278 ± 0.94 ^ab^	0.38 ± 0.01 ^d^
L10	10.8 ± 0.12 ^ab^	137.9 ± 0.58 ^ab^	320 ± 1.25 ^d^	0.32 ± 0.01 ^b^
L15	11.0 ± 0.12 ^ab^	138.7 ± 0.33 ^b^	301 ± 0.47 ^c^	0.34 ± 0.01 ^cd^
L20	10.1 ± 0.17 ^a^	139.8 ± 0.87 ^bc^	270 ± 2.49 ^b^	0.39 ± 0.01 ^d^
L25	10.2 ± 0.12 ^a^	140.3 ± 0.29 ^c^	247 ± 1.25 ^a^	0.44 ± 0.01 ^f^
P10	12.6 ± 0.45 ^d^	138.2 ± 0.75 ^b^	322 ± 1.70 ^d^	0.31 ± 0.00 ^b^
P15	12.1 ± 1.70 ^c^	139.2 ± 0.53 ^b^	300 ± 1.25 ^c^	0.32 ± 0.00 ^bc^
P20	11.9 ± 0.21 ^b^	139.4 ± 0.38 ^bc^	290 ± 2.16 ^b^	0.37 ± 0.01 ^d^
P25	12.1 ± 0.09 ^c^	139.8 ± 0.49 ^bc^	286 ± 0.47 ^b^	0.38 ± 0.01 ^d^

SA—sample, BL—baking loss, BY—bread yield, BV—bread volume, CD—crumb density, C—control bread, CP10–CP25—breads containing, respectively, 10, 15, 20, and 25% chickpea flour, FB10–FB25—breads containing, respectively, 10, 15, 20, and 25% field bean flour; L10–L25—breads containing, respectively, 10, 15, 20, and 25% lentil flour; P10–P25—breads containing, respectively, 10, 15, 20, and 25% pea flour; the values designated by the letters ^a–f^ are significantly different (*p* < 0.05).

**Table 3:** Crumb texture parameters.

SA	CH [N]	CE [-]	CS [-]	CC [-]
C	8.16 ± 0.09 ^a^	0.23 ± 0.00 ^e^	0.88 ± 0.00 ^c^	0.66 ± 0.00 ^e^
CP10	11.04 ± 0.24 ^c^	0.21 ± 0.01 ^e^	0.86 ± 0.01 ^c^	0.54 ± 0.03 ^d^
CP15	12.59 ± 0.16 ^cd^	0.19 ± 0.00 ^d^	0.85 ± 0.01 ^c^	0.46 ± 0.02 ^c^
CP20	15.86 ± 0.45 ^f^	0.18 ± 0.00 ^d^	0.85 ± 0.00 ^c^	0.44 ± 0.01 ^b^
CP25	17.22 ± 0.24 ^g^	0.13 ± 0.00 ^a^	0.79 ± 0.01 ^ab^	0.41 ± 0.00 ^a^
FB10	9.52 ± 0.32 ^b^	0.22 ± 0.00 ^e^	0.85 ± 0.00 ^c^	0.64 ± 0.02 ^e^
FB15	10.36 ± 0.16 ^b^	0.19 ± 0.00 ^d^	0.82 ± 0.00 ^b^	0.60 ± 0.01 ^de^
FB20	11.30 ± 0.21 ^c^	0.17 ± 0.01 ^b^	0.80 ± 0.02 ^ab^	0.54 ± 0.01 ^d^
FB25	12.06 ± 0.08 ^cd^	0.16 ± 0.01 ^b^	0.79 ± 0.00 ^a^	0.49 ± 0.02 ^c^
L10	12.68 ± 0.18 ^d^	0.21 ± 0.01 ^de^	0.86 ± 0.01 ^c^	0.64 ± 0.01 ^e^
L15	13.62 ± 0.12 ^e^	0.20 ± 0.01 ^de^	0.84 ± 0.00 ^b^	0.52 ± 0.03 ^c^
L20	16.77 ± 0.17 ^f^	0.18 ± 0.01 ^cd^	0.79 ± 0.00 ^ab^	0.45 ± 0.02 ^b^
L25	20.40 ± 0.13 ^h^	0.15 ± 0.00 ^a^	0.75 ± 0.01 ^a^	0.42 ± 0.02 ^a^
P10	9.45 ± 0.30 ^b^	0.22 ± 0.01 ^e^	0.84 ± 0.01 ^b^	0.51 ± 0.02 ^c^
P15	10.09 ± 0.08 ^b^	0.20 ± 0.01 ^de^	0.83 ± 0.02 ^b^	0.50 ± 0.00 ^c^
P20	12.30 ± 0.16 ^cd^	0.19 ± 0.01 ^d^	0.82 ± 0.00 ^b^	0.49 ± 0.01 ^c^
P25	13.19 ± 0.17 ^d^	0.16 ± 0.00 ^b^	0.78 ± 0.03 ^a^	0.45 ± 0.00 ^b^

SA—sample, CH—hardness, CE—elasticity, CS—springiness, CC—cohesiveness, C—control bread, CP10–CP25—bread containing, respectively, 10, 15, 20, and 25% chickpea flour, FB10–FB25—bread containing, respectively, 10, 15, 20, and 25% field bean flour; L10–L25—bread containing, respectively, 10, 15, 20, and 25% lentil flour; P10–P25—bread containing, respectively, 10, 15, 20, and 25% pea flour; the values designated by the letters ^a–h^ are significantly different (*p* < 0.05).

**Table 4:** Color of flours and crumb samples.

SA	L* [-]	R* [-]	Y* [-]	ΔE [-]
WF	91.01 ± 0.10 ^D^	0.41 ± 0.02 ^A^	10.01 ± 0.21 ^A^	-
CPF	86.39 ± 0.28 ^C^	2.17 ± 0.01 ^C^	20.17 ± 0.53 ^E^	-
FBF	83.00 ± 0.19 ^B^	0.46 ± 0.03 ^A^	13.54 ± 0.31 ^B^	-
LF	79.92 ± 0.48 ^A^	5.53 ± 0.07 ^D^	16.62 ± 0.19 ^C^	-
PF	82.33 ± 0.19 ^B^	1.56 ± 0.04 ^B^	18.28 ± 0.13 ^D^	
C	71.22 ± 0.78 ^f^	0.12 ± 0.02 ^a^	13.64 ± 0.24 ^a^	-
CP10	67.89 ± 0.26 ^e^	0.49 ± 0.04 ^bc^	16.87 ± 0.22 ^b^	3.0
CP15	64.58 ± 0.34 ^d^	0.82 ± 0.15 ^d^	18.98 ± 1.44 ^c^	7.8
CP20	62.50 ± 0.22 ^d^	1.93 ± 0.04 ^f^	21.48 ± 0.73 ^e^	10.9
CP25	60.77 ± 0.61 ^c^	2.14 ± 0.14 ^f^	22.65 ± 0.41 ^e^	13.1
FB10	66.94 ± 0.11 ^e^	0.32 ± 0.07 ^b^	14.67 ± 0.31 ^a^	3.5
FB15	64.52 ± 0.13 ^d^	0.41 ± 0.02 ^b^	15.85 ± 0.45 ^b^	6.1
FB20	63.53 ± 0.47 ^d^	0.58 ± 0.20 ^bc^	17.98 ± 0.86 ^c^	7.8
FB25	60.63 ± 0.20 ^c^	1.17 ± 0.07 ^e^	20.10 ± 0.41 ^d^	11.4
L10	60.49 ± 0.22 ^c^	1.26 ± 0.23 ^e^	16.82 ± 0.11 ^b^	10.3
L15	57.26 ± 0.12 ^b^	1.49 ± 0.10 ^e^	17.47 ± 0.79 ^bc^	13.6
L20	56.51 ± 0.37 ^b^	2.01 ± 0.22 ^f^	18.85 ± 0.87 ^c^	14.7
L25	52.90 ± 0.09 ^a^	2.46 ± 0.17 ^g^	19.61 ± 0.63 ^d^	18.4
P10	68.59 ± 0.27 ^e^	0.70 ± 0.09 ^c^	16.21 ± 0.87 ^b^	3.5
P15	65.65 ± 0.20 ^d^	0.75 ± 0.09 ^c^	17.82 ± 0.45 ^c^	6.0
P20	63.56 ± 0.13 ^d^	1.18 ± 0.04 ^e^	19.71 ± 0.21 ^d^	8.8
P25	58.53 ± 0.33 ^bc^	2.35 ± 0.21 ^g^	20.34 ± 0.64 ^d^	13.5

SA—sample, L*—lightness, R*—redness, Y*—yellowness, ΔE—total color difference, WF—wheat flour, CPF—chickpea flour, FBF—field bean flour, LF—lentil flour, PF—pea flour, C—control bread, CP10–CP25—bread containing, respectively, 10, 15, 20, and 25% chickpea flour, FB10–FB25—bread containing, respectively, 10, 15, 20, and 25% field bean flour; L10–L25—bread containing, respectively, 10, 15, 20, and 25% lentil flour; P10–P25—bread containing, respectively, 10, 15, 20, and 25% pea flour; the values designated by the different letters ^A–E^ or ^a–g^ are significantly different (*p* < 0.05).

**Table 5:** Basic chemical composition of used flours and obtained breads.

SA	PC [% DM]	AC [% DM]	FB [% DM]	FA [% DM]	CB [% DM]
WF	11.12 ± 0.13 ^A^	0.54 ± 002 ^A^	1.85 ± 0.01 ^A^	0.85 ± 0.01 ^B^	85.64 ± 0.15 ^E^
CPF	22.18 ± 0.03 ^B^	2.86 ± 0.01 ^B^	6.51 ± 0.08 ^D^	4.88 ± 0.02 ^E^	63.57 ± 0.04 ^D^
FBF	36.32 ± 0.02 ^E^	3.87 ± 0.00 ^D^	5.40 ± 0.05 ^B^	1.50 ± 0.02 ^C^	52.91 ± 0.06 ^A^
LF	30.74 ± 0.09 ^C^	4.08 ± 0.02 ^E^	5.60 ± 0.02 ^C^	0.39 ± 0.03 ^A^	59.19 ± 0.07 ^C^
PF	31.13 ± 0.06 ^D^	3.72 ± 0.04 ^C^	7.77 ± 0.06 ^E^	2.39 ± 0.05 ^D^	54.99 ± 0.04 ^B^
CB	11.39 ± 0.04 ^a^	0.86 ± 0.01 ^a^	1.91 ± 0.05 ^a^	1.30 ± 0.02 ^d^	84,54 ± 0.40 ^k^
CP10	12.72 ± 0.08 ^b^	1.18 ± 0.00 ^b^	2.06 ± 0.01 ^bc^	1.62 ± 0.02 ^hi^	82.42 ± 0.09 ^j^
CP15	13.48 ± 0.05 ^d^	1.26 ± 0.00 ^c^	2.12 ± 0.02 ^cd^	1.80 ± 0.02 ^j^	81.34 ± 0.05 ^i^
CP20	14.03 ± 0.06 ^e^	1.39 ± 0.01 ^e^	2.54 ± 0.03 ^h^	2.02 ± 0.02 ^k^	80,02 ± 0.11 ^gh^
CP25	14.47 ± 0.03 ^f^	1.44 ± 0.00 ^f^	2.71 ± 0.03 ^i^	2.12 ± 0.06 ^l^	79.22 ± 0.02 ^e^
FB10	14.22 ± 0.03 ^e^	1.34 ± 0.01 ^d^	1.99 ± 0.02 ^ab^	1.41 ± 0.01 ^e^	81.04 ± 0.05 ^i^
FB15	15.27 ± 0.03 ^h^	1.42 ± 0.01 ^ef^	2.10 ± 0.01 ^bcd^	1.44 ± 0.02 ^ef^	79.76 ± 0.01 ^fg^
FB20	16.29 ± 0.05 ^i^	1.51 ± 0.01 ^h^	2.30 ± 0.02 ^ef^	1.52 ± 0.02 ^fg^	78.32 ± 0.05 ^d^
FB25	18.22 ± 0.02 ^k^	1.70 ± 0.02 ^k^	2.43 ± 0.03 ^gh^	1.63 ± 0.01 ^i^	76.01 ± 0.02 ^a^
L10	13.09 ± 0.05 ^c^	1.30 ± 0.02 ^d^	2.18 ± 0.02 ^de^	1.21 ± 0.02 ^c^	82.22 ± 0.03 ^j^
L15	14.07 ± 0.10 ^e^	1.59 ± 0.00 ^j^	2.74 ± 0.01 ^i^	1.18 ± 0.01 ^bc^	80.42 ± 0.13 ^h^
L20	15.00 ± 0.03 ^g^	1.74 ± 0.01 ^k^	2.82 ± 0.03 ^i^	1.10 ± 0.01 ^ab^	79.34 ± 0.00 ^ef^
L25	16.09 ± 0.13 ^i^	2.10 ± 0.02 ^l^	3.24 ± 0.07 ^j^	1.07 ± 0.02 ^a^	77.50 ± 0.19 ^b^
P10	13.60 ± 0.05 ^d^	1.26 ± 0.00 ^c^	2.32 ± 0.03 ^fg^	1.43 ± 0.02 ^e^	81.39 ± 0.02 ^i^
P15	14.56 ± 0.11 ^f^	1.42 ± 0.00 ^ef^	2.43 ± 0.02 ^gh^	1.52 ± 0.02 ^fg^	80.07 ± 0.10 ^gh^
P20	15.32 ± 0.04 ^h^	1.50 ± 0.01 ^h^	2.71 ± 0.03 ^i^	1.54 ± 0.02 ^gh^	78.93 ± 0.05 ^e^
P25	16.56 ± 0.10 ^j^	1.55 ± 0.00 ^i^	3.22 ± 0.03 ^j^	1.61 ± 0.02 ^hi^	77.06 ± 0.10 ^b^

SA—sample, PC—protein, AC—ash, FB—fiber, FA—fat, CB—carbohydrates, WF—wheat flour, CPF—chickpea flour, FBF—field bean flour, LF—lentil flour, PF—pea flour, C—control bread, CP10–CP25—bread containing, respectively, 10, 15, 20, and 25% chickpea flour, FB10–FB25—bread containing, respectively, 10, 15, 20, and 25% field bean flour; L10–L25—bread containing, respectively, 10, 15, 20, and 25% lentil flour; P10–P25—bread containing, respectively, 10, 15, 20, and 25% pea flour; the values designated by the different letters ^A–E^ or ^a–l^ are significantly different (*p* < 0.05).

**Table 6:** Phenolic content and antioxidant capacity of flours and breads.

SA	TPC [mg GAE g DM^−1^]	EC_50 DPPH_ [mg DM mL^−1^]	EC_50 ABTS_ [mg DM mL^−1^]
WF	0.45± 0.04 ^A^	224 ± 4.06 ^D^	249 ± 3.01 ^E^
CPF	0.85± 0.07 ^B^	67 ± 2.12 ^C^	58 ± 0.72 ^D^
FBF	1.92± 0.08 ^D^	36 ± 0.61 ^B^	33 ± 0.19 ^C^
LF	1.12± 0.05 ^C^	34 ± 1.02 ^B^	29 ± 0.09 ^B^
PF	2.36± 0.11 ^E^	25 ± 0.29 ^A^	22 ± 0.06 ^A^
C	0.46 ± 0.02 ^a^	242 ± 3.68 ^j^	285 ± 9.20 ^j^
CP10	0.43 ± 0.02 ^a^	139 ± 4.24 ^h^	260 ± 10.15 ^i^
CP15	0.47 ± 0.01 ^a^	110 ± 4.50 ^fg^	249 ± 1.34 ^h^
CP20	0.47 ± 0.00 ^a^	106 ± 2.83 ^f^	245 ± 3.47 ^h^
CP25	0.54 ± 0.00 ^b^	96 ± 3.68 ^f^	169 ± 10.74 ^g^
FB10	0.56 ± 0.01 ^b^	107 ± 0.26 ^f^	103 ± 0.23 ^f^
FB15	0.63 ± 0.00 ^c^	104 ± 0.25 ^f^	82 ± 0.63 ^e^
FB20	0.84 ± 0.00 ^de^	71 ± 1.31 ^e^	67 ± 0.42 ^d^
FB25	1.01 ± 0.03 ^e^	57 ± 0.13 ^d^	57 ± 0.07 ^c^
L10	0.55 ± 0.01 ^b^	236 ± 4.41 ^j^	150 ± 1.28 ^g^
L15	0.61 ± 0.02 ^c^	158 ± 0.00 ^i^	124 ± 0.58 ^g^
L20	0.75 ± 0.00 ^d^	138 ± 5.65 ^h^	110 ± 7.59 ^f^
L25	0.85 ± 0.00 ^de^	117 ± 2.05 ^g^	82 ± 2.06 ^e^
P10	0.77 ± 0.00 ^d^	49 ± 1.00 ^c^	58 ± 0.63 ^c^
P15	0.99 ± 0.00 ^e^	40 ± 0.07 ^b^	43 ± 0.04 ^b^
P20	1.14 ± 0.01 ^e^	36 ± 0.08 ^a^	35 ± 0.07 ^a^
P25	1.37 ± 0.01 ^f^	35 ± 0.08 ^a^	33 ± 0.11 ^a^

SA—sample, TPC—phenolic content, EC50_DPPH_, EC50_ABTS_—antioxidant capacity against DPPH and ABTS, respectively, WF—wheat flour, CPF—chickpea flour, FBF—field bean flour, LF—lentil flour, PF—pea flour, C—control bread, CP10–CP25—bread containing, respectively, 10, 15, 20, and 25% chickpea flour, FB10–FB25—bread containing, respectively, 10, 15, 20, and 25% field bean flour; L10–L25—bread containing, respectively, 10, 15, 20, and 25% lentil flour; P10–P25—bread containing, respectively, 10, 15, 20, and 25% pea flour; the values designated by the different letters ^A–E^ or ^a–j^ are significantly different (*p* < 0.05).

**Table 7:** Findings from the sensory assessment of obtained breads.

SA	APP	SM	TA	TEX	COL	OA
C	8.5 ± 0.50 ^d^	8.5 ± 0.92 ^d^	8.7 ± 0.64 ^e^	8.7 ± 0.64 ^f^	8.1 ± 0.83 ^e^	8.5 ± 0.50 ^d^
CP10	7.5 ± 0.50 ^c^	8.2 ± 0.87 ^d^	7.0 ± 0.63 ^d^	8.2 ± 0.60 ^e^	8.0 ± 0.89 ^e^	7.7 ± 0.79 ^c^
CP15	7.3 ± 0.46 ^bc^	5.3 ± 1.10 ^b^	5.8 ± 0.87 ^c^	5.5 ± 1.02 ^c^	5.4 ± 0.66 ^c^	5.8 ± 0.75 ^b^
CP20	6.5 ± 0.50 ^b^	5.1 ± 1.04 ^b^	5.8 ± 0.87 ^c^	5.4 ± 1.11 ^c^	5.1 ± 1.22 ^c^	5.5 ± 0.75 ^b^
CP25	5.4 ± 1.02 ^a^	3.4 ± 1.36 ^a^	3.5 ± 1.02 ^a^	2.8 ± 0.75 ^a^	4.1 ± 0.83 ^b^	3.8 ± 0.64 ^a^
FB10	7.5 ± 0.50 ^c^	8.0 ± 0.77 ^c^	8.3 ± 0.64 ^de^	8.0 ± 0.45 ^e^	8.1 ± 0.83 ^e^	7.8 ± 0.83 ^c^
FB15	7.5 ± 0.50 ^c^	7.7 ± 0.78 ^c^	7.6 ± 0.49 ^d^	7.0 ± 0.89 ^d^	6.8 ± 0.75 ^d^	7.2 ± 0.77 ^c^
FB20	6.9 ± 0.30 ^b^	5.2 ± 1.17 ^b^	5.7 ± 0.78 ^c^	5.5 ± 1.02 ^c^	5.3 ± 1.00 ^c^	5.6 ± 0.72 ^b^
FB25	5.8 ± 0.40 ^ab^	4.5 ± 1.50 ^ab^	4.2 ± 0.75 ^ab^	3.8 ± 1.46 ^ab^	4.8 ± 0.75 ^b^	4.6 ± 0.56 ^ab^
L10	8.3 ± 0.46 ^d^	8.4 ± 0.80 ^d^	8.5 ± 0.67 ^e^	8.3 ± 0.90 ^ef^	7.5 ± 0.92 ^d^	8.1 ± 0.70 ^cd^
L15	7.4 ± 0.49 ^c^	8.0 ± 0.77 ^cd^	8.3 ± 0.64 ^de^	8.0 ± 0.45 ^e^	7.5 ± 0.67 ^d^	7.7 ± 0.82 ^c^
L20	6.8 ± 0.40 ^b^	5.2 ± 1.17 ^b^	5.6 ± 0.80 ^c^	5.4 ± 1.11 ^c^	5.3 ± 1.00 ^c^	5.6 ± 0.70 ^b^
L25	5.6 ± 1.11 ^a^	3.6 ± 1.56 ^ab^	3.6 ± 1.11 ^a^	2.9 ± 0.83 ^a^	4.2 ± 0.87 ^b^	4.0 ± 0.73 ^a^
P10	7.8 ± 0.40 ^c^	8.1 ± 0.30 ^cd^	8.4 ± 0.49 ^de^	8.1 ± 0.30 ^e^	8.1 ± 0.30 ^e^	7.9 ± 0.58 ^cd^
P15	6.9 ± 0.30 ^bc^	7.6 ± 0.66 ^c^	7.5 ± 0.67 ^d^	7.6 ± 0.66 ^d^	7.3 ± 0.64 ^d^	7.3 ± 0.70 ^c^
P20	6.3 ± 0.46 ^ab^	4.8 ± 1.72 ^b^	4.6 ± 1.80 ^b^	4.3 ± 1.19 ^b^	3.8 ± 1.54 ^ab^	4.7 ± 1.12 ^ab^
P25	5.3 ± 1.10 ^a^	3.1 ± 1.58 ^a^	3.3 ± 1.19 ^a^	2.1 ± 0.70 ^a^	3.1 ± 0.70 ^a^	3.4 ± 0.66 ^a^

SA—sample, APP—appearance, SM—smell, TA—taste, TEX—texture, COL—color, OA, overall acceptability, WF—wheat flour, CPF—chickpea flour, FBF—field bean flour, LF—lentil flour, PF—pea flour, C—control bread, CP10–CP25—bread containing, respectively, 10, 15, 20, and 25% chickpea flour, FB10–FB25—bread containing, respectively, 10, 15, 20, and 25% field bean flour; L10–L25—bread containing, respectively, 10, 15, 20, and 25% lentil flour; P10–P25—bread containing, respectively, 10, 15, 20, and 25% pea flour; the values designated by the different letters ^a–f^ are significantly different (*p* < 0.05).

## Data Availability

The original contributions presented in the study are included in the article, further inquiries can be directed to the corresponding author.
